# Establishing a National Cardiopulmonary Resuscitation Program in Indian High schools and colleges: An expert collaborative call to action and implementation model

**DOI:** 10.1016/j.ipej.2025.11.011

**Published:** 2025-12-03

**Authors:** Dhanunjaya Lakkireddy, Jiaqi Mi, Aditya Kapoor, Aashish Katapadi, Jalaj Garg, Rakesh Gopinathannair, Deepak Padmanabhan, Sana M. Al-Khatib, Jagmeet P. Singh, Vanita Arora, Anil Saxena, Christina Y. Miyaki, Eduardo B. Saad, Suresh Allamshetty, Anoop Gupta, Rakesh Yadav, Suchit Mazumdar, Nitish Naik, David S. Frankel, Ashish Nabar, Yash Lokhandwala, Calambur Narasimhan, Daniel P. Morin, Jodie L. Hurwitz, Kenneth A. Ellenbogen, Ajay M. Naik, Mina K. Chung

**Affiliations:** a Kansas City Heart Rhythm Institute, Overland Park, KS, USA; b Sanjay Gandhi Postgraduate Institute of Medical Sciences, Lucknow, India; c HCA Midwest Health, Overland Park, KS, USA; d Loma Linda University Health, Loma Linda, CA, USA; e Sri Jayadeva Institute of Cardiovascular Sciences and Research, Bengaluru, India; f Duke University School of Medicine, Durham, NC, USA; g Massachusetts General Hospital, Boston, MA, USA; h Indraprastha Apollo Hospital, New Delhi, India; i Fortis Escorts Heart Institute, New Delhi, India; j Baylor College of Medicine, Houston, TX, USA; k Beth Israel Deaconess Medical Center, Harvard Medical School, Boston, MA, USA; l Apollo Hospitals, Health City, Arilova, Vishakhapatnam, Andhra Pradesh, 530040, India; m EPIC Multispecialty Hospital, Ahmedabad, Gujarat, India; n All India Institute of Medical Sciences, New Delhi, India; o Apollo Gleneagles Hospitals, Kolkata, West Bengal, India; p Hospital of the University of Pennsylvania, Perelman School of Medicine at the University of Pennsylvania, Philadelphia, PA, USA; q Seth GS Medical College, KEM Hospital, Achary Donde Marg, Parel, Mumbai, Maharashtra, India; r LTMG Hospital, Sion, Mumbai, India; s AIG Institute of Cardiac Sciences, Gachibowli, India; t Mayo Clinic College of Medicine & Science, Rochester, MN, USA; u North Texas Heart Center, Dallas, TX, USA; v Virginia Commonwealth University, Richmond, VA, USA; w Sanjay Gandhi PGIMS, Lucknow, India; x Cleveland Clinic, Cleveland, OH, USA

**Table:** ABBREVIATIONS

**AED**	Automated external defibrillator
**AIIMS**	All india institute of medical sciences
**AHA**	American heart association
**BLS**	Basic life support
**CARES**	Cardiac arrest registry to enhance survival
**CAE**	Cyanoacrylate adhesive closure
**CARD**	Cardiac arrest registry database
**CIMS**	Care institute of medical sciences
**COLS**	Compression-only life support
**CPR**	Cardiopulmonary resuscitation
**CSR**	Corporate social responsibility
**EMS**	Emergency medical services
**ERC**	European resuscitation council
**HB**	House bill
**HRS**	Heart Rhythm Society
**ILCOR**	International liaison committee on resuscitation
**IRB**	Institutional review board
**IAP**	Indian Academy of Pediatrics
**MOHFW**	Ministry of health and family welfare (India)
**MOE**	Ministry of education (India)
**NCC**	National cadet corps
**NGO**	Non-governmental organization
**NMA**	Network meta-analysis
**NSS**	National service scheme
**OHCA**	Out-of-hospital cardiac arrest
**PAROS**	Pan-Asian resuscitation outcomes study
**PE**	Physical education
**SCD**	Sudden cardiac death
**STEMI**	St-elevation myocardial infarction
**WHO**	World health organization

## 1 Introduction

Sudden cardiac arrest (SCA)—defined as death occurring within an hour of symptom onset when witnessed, or within 24 h when unwitnessed—accounts for up to 20 % of all deaths worldwide [1,2]. Even in high-income countries, the incidence of SCA is estimated at 36–60 per 100,000 individuals [3,4]. Despite advances in resuscitation systems of care, survival after SCA remains below 10 % in most regions, with marked disparities across sex, race, and gender [5]. Multiple cardiovascular societies have therefore recognized SCA as a major public health concern and underscored the need for improved surveillance and prevention strategies through early cardiopulmonary resuscitation (CPR) and the use of automated external defibrillators (AEDs) [6]. Immediate CPR approximately doubles the odds of survival, yet bystander-initiated CPR rates range from 2.9 to 80.3 % worldwide and are often below 10 % in low-to middle-income countries [7].

Although national data in India remain limited, estimates from South India suggest that SCA accounts for approximately 10.3 % of all deaths, making it a leading cause of mortality [8]. However, bystander-initiated CPR rates remain extremely low, ranging from 1.3 to 9.8 %, and only one population-based study reported an incidence of 27.8 % [2,9,10]. In that same cohort, merely 12.5 % of emergency medical services (EMS) personnel were CPR-trained—highlighting that poor outcomes stem not only from lack of public awareness but also from inadequate training within the healthcare system. The absence of a unified national cardiac arrest registry further constrains accurate surveillance and evidence-based policymaking. In contrast, registries such as CARES (Cardiac Arrest Registry to Enhance Survival) in the United States and PAROS (Pan-Asian Resuscitation Outcomes Study) in the Asia-Pacific region have been instrumental in guiding system-wide improvements [11]. Without similar infrastructure, India faces critical gaps in understanding and addressing the true burden of sudden cardiac death (SCD).

India's challenges mirror those of other low- and middle-income countries (LMICs), where fragmented EMS systems, cultural hesitancy to intervene, and limited AED access contribute to persistently poor outcomes [[12], [13], [14]]. These systemic barriers reinforce the urgency for a coordinated national strategy for CPR and AED training that extends beyond healthcare settings into the community. School-based resuscitation programs represent a sustainable and socially transformative approach. By leveraging India's vast educational network, such programs can build a self-renewing pool of trained responders, normalize life-saving behaviors from a young age, and embed CPR awareness within broader societal values.

Embedding CPR and AED training into the educational system also carries broader national significance. As India advances toward the Vikasit Bharat 2047 vision, improving public health literacy and community readiness becomes a prerequisite for a resilient and empowered society. Equipping the country's youth, its greatest demographic asset, with lifesaving skills represents a tangible and strategic step toward this long-term national goal. This initiative reflects how health preparedness, education, and civic responsibility intersect to drive India's development agenda.

This expert consensus statement reviews the current landscape of CPR and AED education in India, outlines the core competencies required for a standardized curriculum, and proposes a scalable, multi-phase implementation framework. The recommendations aim to inform national policy, guide stakeholder collaboration, and ultimately improve survival from out-of-hospital cardiac arrest (OHCA) across India and other resource-limited settings globally.

## 2 Prior CPR program experiences

Improving the CPR and AED use rate among the general population relies on the implementation of programs that effectively educate and instill confidence in performing life-saving interventions. Accordingly, numerous efforts worldwide are underway to improve bystander-initiated CPR. Several countries have also successfully implemented national programs, which should be reviewed to create a similar program in India [15].

Several countries with CPR training programs focus on the high school population, though middle school and collegiate students are often also targeted, for reasons discussed below. In Europe, several organizations, such as the European Parliament and the World Health Organization, encourage national legislation to recommend CPR training in secondary schools [16,17]. This has led to an increase in the number of programs. In 2013, four out of 16 European countries had made CPR training an official learning outcome, and 20 states in the US had mandated CPR training for high school students as a graduation requirement. Currently, CPR training is mandated in several European countries, such as Denmark, France, and the United Kingdom, and is recommended but not required in 23 others[[18], [19], [20]]. Other countries, such as Germany, Ireland, the Netherlands, and Norway, recommend CPR training but do not have laws that mandate it. For example, Spain has laws that mandate first-aid training, which ideally includes CPR training for students and staff, but not CPR itself [21]. In 2025, the US has 43 states with mandated CPR training, aligned with guidelines from the American Heart Association (AHA) and American Red Cross, for high school graduation [22]. Similar programs, which vary by territory, exist in Australia, and rapidly expanding national and local programs are also being implemented in China. However, even when CPR is mandated, it does not always result in full implementation. There may be a lack of standardized training, as not all training programs include AED education and training, hands-on CPR practice, and repetition. In a recent survey of high school students in the US, a vast majority of students felt poorly prepared to perform CPR or use AEDs [23].

One of the most transformative efforts in global CPR education is the Kids Save Lives campaign, jointly launched by the World Health Organization (WHO) and the International Liaison Committee on Resuscitation (ILCOR) [20,24]. The initiative calls for all schoolchildren aged 12 years and older to receive at least 2 h of CPR training each year, with the goal of improving survival from sudden cardiac arrest. It focuses not only on teaching skills but also on nurturing the motivation and confidence needed for children to take action. Students are taught to recognize unresponsiveness and abnormal breathing, call for emergency medical services, and begin compression-only CPR with proper rate and depth, using the straightforward CHECK–CALL–COMPRESS approach. The program envisions CPR education as a progressive process beginning early in life [24]. Foundational concepts can be introduced as early as age four, enabling children to recognize an emergency and alert EMS by age six. From around age twelve, students begin hands-on chest compression practice with periodic refreshers—brief 5-min sessions every few months have been shown to significantly reinforce skill retention [25]. By approximately age fourteen, most adolescents have sufficient physical capacity to deliver effective ventilations [26]. AED education follows a similarly stepwise approach; studies demonstrate that children as young as five to seven years can follow AED voice instructions and safely deliver a shock, though the highest accuracy and confidence are observed among those aged thirteen and older [26]. Taken together, the Kids Save Lives program establishes a structured and developmentally appropriate framework that empowers children to become confident first responders within their communities.

Another important public education initiative is the “Leave My Tongue Alone” campaign, which challenges the persistent myth that cardiac arrest victims “die because they swallow their tongue.” This misconception, often seen in athletic settings, diverts bystanders from initiating chest compressions while they attempt to open the mouth or reposition the tongue. Analyses of on-field cardiac arrests captured on video have shown that such delays in CPR initiation are linked to poorer survival and neurologic outcomes [27,28]. The campaign reinforces the essential message that survival depends on early recognition, immediate compressions, and rapid defibrillation—not airway manipulation. Incorporating this myth-busting education into school and community CPR programs can improve public understanding and bystander readiness.

Globally, countries that have integrated cardiopulmonary resuscitation (CPR) education into school curricula have demonstrated substantial improvements in bystander response and survival following cardiac arrest. Only a few nations, such as Denmark, Belgium, France, Portugal, Italy, and the United Kingdom, have implemented programs that fully align with international recommendations [29]. Among these, Denmark stands out, with studies showing marked increases in bystander-initiated CPR and significant improvements in both hospital admission and 30-day survival rates after the introduction of comprehensive school- and community-based initiatives [30]. However, even within these countries, considerable heterogeneity in implementation persists [31]. Recent data from Denmark highlight that, despite 19 years of mandatory legislation, fewer than 50 % of schools have implemented CPR training, underscoring that laws alone are insufficient without enforcement and monitoring. In 2024, surveys revealed that while 72 % of Canadian rural schools had AEDs, only 24 % provided CPR training to students, highlighting the gap between device availability and practical skill-building.

Building on these global experiences, India can adapt proven models from Denmark, Norway, and Japan to its unique demographic and institutional context. By leveraging its extensive educational infrastructure, India has the potential to establish a sustainable, large-scale framework for CPR awareness and training—creating a self-renewing generation of lifesavers and embedding cardiac arrest preparedness as a core element of public health education.

## 3 State of CPR and AED access in India

SCA represents a significant public health burden in India, with estimates suggesting that more than 700,000 people die annually due to it [9,32].Survival from out-of-hospital cardiac arrest (OHCA) remains extremely poor, with survival to hospital discharge reported at less than 2–3 % in Indian literature [9]. This is in stark contrast to the 10–20 % survival rates observed in countries with mature community resuscitation systems [33,34]. However, only 2–6.5 % of urban laypersons can perform CPR, with less than 15 % having ever received formal training [35]. Even worse, bystander-initiated CPR rates in India remain consistently low, at 1.3–9.8 %, compared to 40–60 % in other high-income countries [[36], [37], [38]].Additionally, willingness to perform CPR is often limited by fears of causing harm, medicolegal concerns, and cultural hesitations – particularly those related to gender. While healthcare providers typically receive training, the dissemination of CPR skills into schools, workplaces, and communities has been minimal. Notably, CPR instruction is absent from mainstream high school and college curricula.

AED access is even more concerning. AEDs are rarely available outside select airports, luxury hotels, and corporate campuses. Most public schools, colleges, and community centers lack access to AEDs, leaving large swaths of the population unprotected. Costs remain a major barrier, with imported AEDs priced beyond the reach of most institutions. Maintenance concerns, particularly battery and defibrillator pad replacement, as well as risks of vandalism, further limit adoption [39]. Equally problematic is the lack of public familiarity with AEDs. Even when devices are present, few individuals feel comfortable using them. Recent Indian survey evidence reports that only about 11.5 % of the public have ever received training in CPR or AED use, and practical knowledge or confidence to use an AED in an emergency is described as alarmingly deficient [40].

The current state of CPR and AED access in India remains fragmented, inequitable, and insufficient to meet the country's growing burden of sudden cardiac arrest (SCA). The absence of a national legislative framework mandating CPR education or public AED deployment, coupled with the lack of a unified SCA registry or centralized emergency medical services (EMS) system, poses major structural challenges. Existing EMS networks are often fragmented and under-resourced; in one study, 41.5 % of out-of-hospital cardiac arrest (OHCA) patients arrived at the hospital by non-ambulance means, and only 12.5 % of ambulances had personnel trained in basic life support [10]. Response times frequently exceed 15–20 min, particularly in semi-urban and rural areas, reducing the chances of timely intervention [41]. Consequently, bystander action becomes critical—yet only 9.8 % of patients received bystander CPR, and AED use was reported in less than 1 % of cases [38,39]. In a survey of more than 1800 university students, fewer than one-third had prior CPR training, and only 12–17 % were familiar with hands-only CPR or AED use [42].

While several non-governmental organizations have launched CPR awareness programs, their impact has largely remained localized and unstandardized. The ReviveHeart Foundation (RHF), Mumbai, a not-for-profit initiative founded by India's leading cardiologists and healthcare professionals, has been conducting CPR awareness and training sessions across India since 2017, along with the National Sudden Cardiac Arrest Awareness Campaign since 2021. Under its flagship, more than 1.5 million citizens, including students, healthcare workers, police personnel, and the general public, have been trained in CPR through collaborations with over 150 schools, universities, the Indian Railways, police and armed forces, and corporate partners. The foundation's digital outreach has surpassed 50 million impressions across media and social platforms. RHF has also piloted AED installations in select hospitals, malls, and public venues, while Rotary organizations have supported similar efforts in metro stations in Mumbai.

**Fig. 1:**
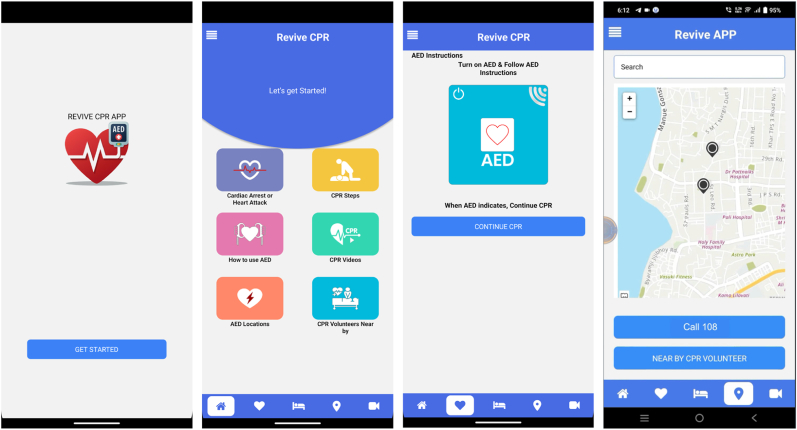
Interface and Key Functions of the Revive CPR App The Revive CPR App provides an accessible, stepwise digital platform to support public CPR readiness in India. The app includes modules on recognizing cardiac arrest, hands-only CPR steps, AED operation guidance, instructional videos, and a geolocation feature that identifies nearby AEDs and trained CPR volunteers. It also offers one-touch emergency activation (Call 108), helping bridge the time gap before EMS arrival. These features illustrate how mobile technology can enhance community preparedness and support national CPR and AED initiatives.

National policy developemnts have begun to create momentum as well. The National Education Policy (NEP) 2020 promotes experiential and skills-based learning, providing an opportunity to embed CPR education within formal curricula. Similarly, initiatives such as the Compulsory Training of CPR in Schools Bill (2019) and the NBEMS Nationwide CPR Awareness Program represent important first steps. To create lasting impact, however, these initiatives must be unified within a national policy framework that ensures broad-based training, systematic AED deployment, and culturally sensitive public engagement. In addition, digital tools such as the Revive CPR App offer an accessible platform for stepwise CPR guidance, training reinforcement, and public engagement. The app's widespread use underscores the potential of mobile technology to enhance community readiness, and its icon is highlighted within the manuscript figures to improve visibility and adoption (Fig. 1).

Despite this progress, most initiatives remain geographically limited and lack sustained infrastructure support. Several pilot programs, including community based hands only CPR trainings, have demonstrated clear improvements in knowledge, confidence, and willingness to act, yet their impact has not scaled nationally [43,44]. These programs remain geographically limited and lack sustained infrastructure support across states. These findings reinforce the need for India to design policies that combine AED deployment with mandated, assessed CPR education.

## 4 Rationale for targeting high school and college students

**Fig. 2:**
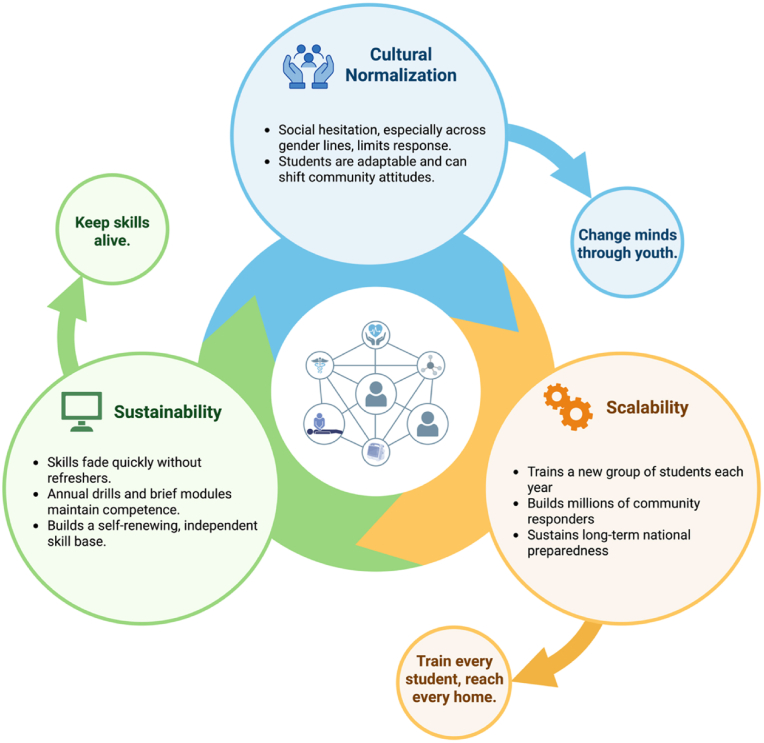
Gaps addressed by targeting high school and college students. Integration of CPR and AED education in schools addresses three key barriers: scalability—training each student cohort to expand responder reach; sustainability—embedding annual refreshers to maintain competence; and cultural normalization—empowering youth to overcome social hesitation and reshape community attitudes. Together, these elements create a self-renewing, nationwide network of capable responders.

India is often described as a country of young people, with one of the largest youth populations in the world. This demographic dividend represents a unique national asset that can be channeled toward community readiness and lifesaving capacity. Training adolescents and young adults in CPR and AED use leverages this demographic strength to build a resilient and empowered future workforce. With more than 250 million students enrolled in secondary schools and universities, this demographic represents an unparalleled opportunity to embed lifesaving skills across society [45]. Training adolescents and young adults in CPR and AED use leverages this demographic strength to build a resilient and empowered future workforce. Targeting high school students aligns with recommendations by the International Liaison Committee on Resuscitation, made in 2003 [46]. The addition of college students offers an added advantage. Training high school and college students in CPR and AED use simultaneously addresses three critical gaps (Fig. 2): scalability, sustainability, and cultural normalization.

First, the scalability of school-based training is unmatched. Every year, millions of young Indians graduate from secondary schools and universities. If CPR and AED education is integrated into curricula, each graduating class adds a fresh cohort of trained individuals into society. Over a decade, this approach could create tens of millions of potential responders, distributed across every state, district, and community. Unlike sporadic training campaigns, this educational strategy ensures steady expansion of the trained population.

Second, sustainability is inherently built into the school and college model. CPR skills are prone to decay within months if not refreshed. Embedding training into the academic calendar—through annual drills, refresher modules, and practical assessments—ensures that students not only learn but retain competence. As long as educational institutions maintain this structure, India will have a self-renewing skill base, with no reliance on short-term workshops or external funding cycles.

Third, cultural normalization is best achieved through youth. Social hesitation—especially around performing chest compressions on strangers or across gender lines—remains a barrier in India. Students, however, are highly adaptable, open to new learning, and capable of reshaping community attitudes. When students bring CPR knowledge into their homes and peer groups, they extend its influence beyond the classroom. Moreover, young people are particularly adept at leveraging digital media to spread awareness, further amplifying the societal impact.

When students apply their knowledge of CPR in their homes and peer groups, they extend its influence beyond the classroom. Moreover, young people may leverage digital media to spread awareness and amplify societal impact. Global experiences support this multiplier effect. In Singapore, the *MyResponder* smartphone application mobilizes trained laypersons, including students, to spread awareness to communities. In Japan, widespread school CPR education since the early 2000s has been credited with improving bystander CPR rates nationwide [47]. Similarly, studies from the US have demonstrated that 70 % of students trained in CPR are more likely to teach peers and family members, thereby creating a ripple effect of lifesaving knowledge [48].

AED placement in universities creates opportunities not only for students but also for the surrounding communities, as campuses often host thousands of students, staff, and visitors daily, where AED deployment can have a direct impact on outcomes. College students trained in AED use can respond effectively both on campus and in their neighborhoods, bridging the critical time gap before EMS arrives. International experience confirms the impact of AED placement on campuses. In US universities, AED programs have reported survival rates exceeding 60–70 % for witnessed arrests on campus [49]. Such data strengthen the argument for prioritizing AED deployment in Indian colleges as community hubs.

## 5 Building a sustainable CPR/AED knowledge base in India

Although India has witnessed several short-term campaigns and isolated AED donations, these efforts have not translated into measurable improvements in OHCA survival [37,50]. The primary reason is the lack of sustainability. Without continuity, retraining, and integration into larger systems, enthusiasm generated by one-off initiatives dissipates quickly. Devices often remain unused or non-functional.

Existing efforts to decrease SCA in India have primarily been led by non-governmental organizations, hospitals, or corporate social responsibility initiatives [50]. While they demonstrate feasibility, they are fragmented, geographically restricted, and reliant on short-term funding. For example, mass training workshops may reach hundreds of individuals, but without built-in retraining, skills tend to fade over time. Without integration into school or college curricula, their reach remains limited. Similarly, donations of AEDs to institutions often occur without training programs or maintenance support, resulting in devices that are non-functional when needed.

The need for sustainable programs is evident from the nature of CPR skills and the technical requirements of AEDs. Skills in CPR deteriorate rapidly, with a measurable decline within 6–12 months if not reinforced [51]. AEDs, likewise, require ongoing maintenance and oversight; pads and batteries must be replaced regularly, and routine checks are needed to ensure readiness [52]. Without mechanisms for refresher training and device maintenance, India risks perpetuating the problem of “AEDs in closets” and unskilled responders. Fragmented and short-lived initiatives cannot meaningfully reduce OHCA mortality. For meaningful change, CPR and AED initiatives in India must be designed to emphasize continuity, renewal, and inclusivity [53].

This can be assisted by technology and innovation. Mobile applications that map AEDs, alert trained responders and provide step-by-step instructions can support lay rescuers in real time. Low-cost manikins and simulation tools, developed locally, can allow schools to conduct training without prohibitive costs. Mobile applications and technology may also facilitate reach of training into rural populations and address cultural hesitations through gender-sensitive and language-appropriate education.

A sustainable program – anchored in legislation, education, community ownership, ongoing training, and evaluation – is the only path forward and depends on a few key factors. 1) Policy support is essential. Enduring progress requires legislative backing, including mandates for CPR education in schools and colleges, as well as requirements for the placement of AEDs in educational institutions [54]. 2) Integration into education is critical. Embedding CPR training within the high school and college curriculum ensures that each new generation of graduates is trained. 3) Community ownership must be emphasized. Teachers, students, and community leaders can spread skills beyond campuses into families and neighborhoods. 4) Recurrent retraining models are necessary to prevent skills decay [55,56]. 5) Finally, strong monitoring systems are required, including data collection on training progress, AED functionality, bystander CPR rates, and survival outcomes [52].

Again, this is confirmed by global lessons. The experience from Denmark and Canada is discussed above. Most recently, the United States passed the HEARTS Act, which charges development of CPR and AED training and upkeep in schools; if funded, this may demonstrate how long-term governmental investment can accelerate toward improving SCD outcomes and underpin sustainability. Non-governmental organizations, such as the Heart Rhythm Society and the American Heart Association, and corporate partners are also stepping up efforts to guide and fulfill the mandate.

## 6 A template for governmental programs

**Fig. 3:**
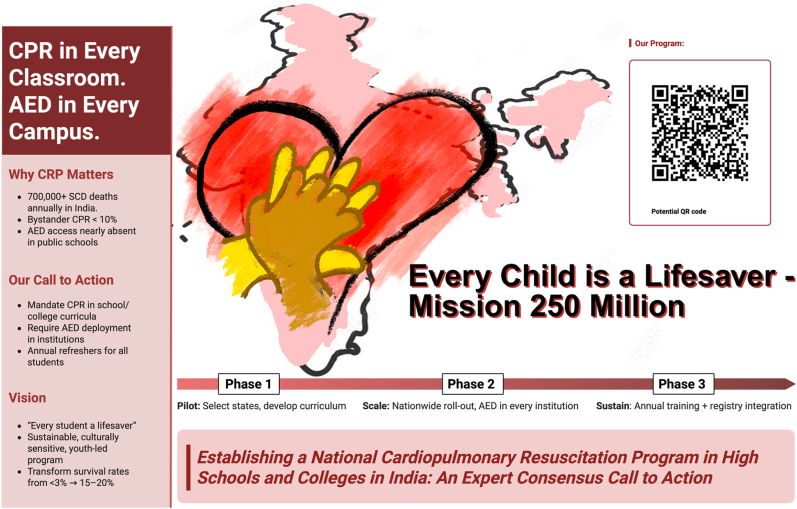
Poster for CPR and AED campaign.

The preceding sections have highlighted that the burden of SCA and OHCA in India is staggering, primarily due to the absence of widespread CPR and AED use. To address this, India requires a national strategy that embeds lifesaving skills within society at scale. The *Kids Save Lives* campaign, endorsed by the WORLD HEALTH ORGANIZATION, European Resuscitation Council, and Indian Resuscitation Council, provides a tested framework that has been implemented in some form in various countries [57,58]. Herein, we outline a ready-to-implement template based on *Kids Save Lives* for governmental action, proposed as the *HeartSafe India Act – Jeevan Rakshak Abhiyaan* (**Central Illustration)***,* accompanied by a parallel public-facing campaign – *Every Child is a Lifesaver – Mission 250 million* (Fig. 3).

The *HeartSafeIndia Act* would mandate: 1) CPR and AED training as a requirement in national and state school curricula, 2) installation and maintenance of AEDs in all secondary schools, colleges, and public universities, 3) creation of a national CPR/AED registry integrated with EMS dispatch, and 4) establishment of a national monitoring council to track compliance and outcomes. The Act would earmark dedicated funding through a public–private partnership model and corporate social responsibility (CSR) contributions, ensuring equity across urban and rural areas. To ensure accountability, schools should be required to submit annual CPR/AED training and audit reports to the Ministry of Education and Health. This approach mirrors international best practices [59].

The public-facing campaign should be launched with mass and social media, and celebrity endorsements to normalize CPR and AED use across Indian society. Periodic national CPR days, synchronized drills, and competitions among schools could reinforce cultural acceptance and create visibility. Most importantly, the program should establish clear national performance targets, aiming to achieve bystander CPR rates above 30 to 40 percent and AED use greater than 10 percent within five years, consistent with international recommendations from the AHA and ILCOR and results observed in leading resuscitation systems worldwide [37,60].

### 6.1 Legislative and policy framework

Legislation provides the backbone of any successful public health intervention. In India, the federal and state governments must collaborate to establish laws that mandate CPR and AED education for all secondary school and undergraduate students. Given that the National Education Policy prioritizes experiential and skills based learning, CPR and AED training should be formally incorporated into the NEP as an essential competency. Embedding this initiative within existing policy frameworks ensures long term sustainability and consistent national implementation.

• Mandatory CPR and AED education in all schools from 6th grade onwards. Training must not be limited to classroom lectures – a minimum of 2 h of hands-on training should be required yearly.
• Specify minimum training requirements, with graduation requirements, integration into existing curriculum, and annual refreshers before secondary school completion. Training should start at 12 years, expanding to AED use by 14 years.
• Stipulate instructor certification to guarantee teaching quality. A national CPR instructor registry should be created, mandating recertification every 2–3 years, modeled after the AHA and European Resuscitation Council standards.
• Include Good Samaritan protection and indemnity clauses for teachers and students to provide aid. India already has an established Good Samaritan Law that protects bystanders who render aid during emergencies. Explicitly reinforcing this legal protection within CPR training can help reduce fear of liability and increase willingness to intervene during cardiac arrest.
• Mandate uniform standards across states in a Federal-State partnership, while allowing for cultural and linguistic adaptation.
• Materials must be provided in multiple Indian languages and adapted for schools serving rural, low-literacy, and special-needs populations.
• Specify explicit funding clauses that allocate resources for manikins, AEDs, training kits, and digital tools.
• Require annual reporting of CPR/AED training activities by schools to the Ministry of Education and Health, with audits by state-level inspectors. This can be used to create a public National CPR Report Card, published annually.
• Establish a national CPR and AED training registry, linked to EMS dispatch, to improve real-time use of devices and mobilize trained responders.
• Target more than 30 % bystander CPR within 5 years, more than 50 % AED availability in schools, and more than 10 % AED use in OHCA by 2030.

To ensure equitable and universal access, these mandates must apply to all categories of educational institutions, including government funded schools, municipal aided schools, private schools, and deemed universities. Incorporating the full spectrum of institutions prevents disparities in training access and promotes national consistency.

### 6.2 Standardized curriculum development

**Fig. 4:**
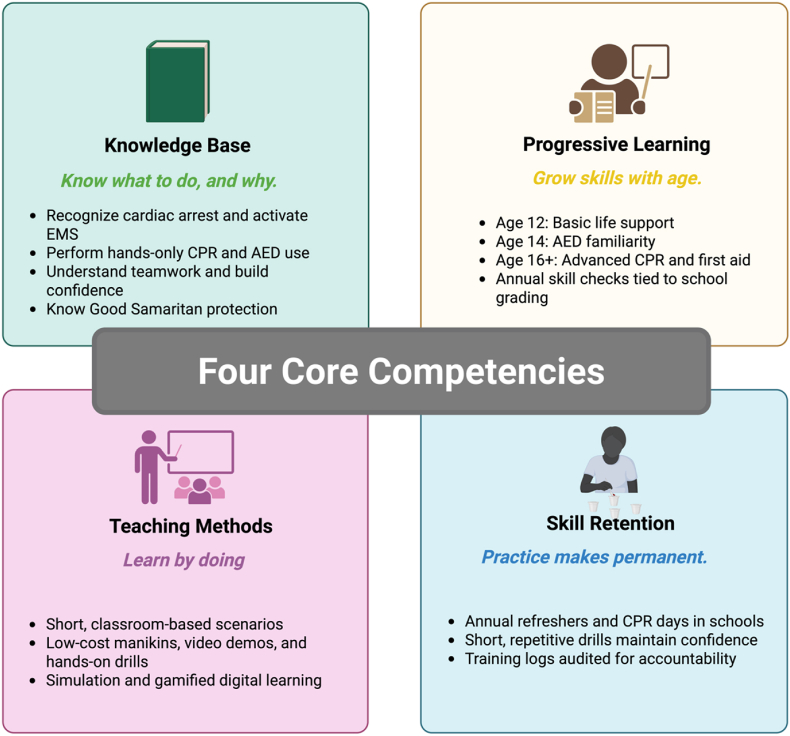
Core competencies for a standardized curriculum. A structured school-based CPR/AED program builds competency across four domains: knowledge base (recognition, response activation, and confidence), progressive learning (age-appropriate, skills-based milestones), teaching methods (interactive, low-cost, and simulation-driven instruction), and skill retention (repetitive practice and performance audits). These competencies form the foundation for a sustainable, nationwide resuscitation training framework.

For mandates to succeed, a standardized national curriculum is essential. This curriculum must be adapted to accommodate India's linguistic diversity and cultural sensitivities, while remaining anchored in internationally recognized guidelines. Findings from the U.S. scoring study emphasize that vague or poorly defined mandates undermine effectiveness [23]. At the same time, a standardized curriculum must balance simplicity with core competencies (Fig. 4).

• Students should be able to recognize SCA, activate emergency services, perform high-quality hands-only CPR, and use an AED safely and effectively.
• Mandate both practical skills demonstration and recurrent training. Scenario-based drills are particularly important for developing confidence in real-world applications, as previous survey results have suggested that fewer than half of US high school students knew the correct compression rate or depth. At least 2 h of CPR training should be performed annually, starting at age 12.
• Education on medicolegal protections should also be included to address common fears about liability or harm.
• Competency assessments should be incorporated into academic grading or graduation requirements.
• Developed by the Indian Resuscitation Council in collaboration with the European Resuscitation Council and AHA, and tailored for India.
• Embed “train-the-trainer” modules for teachers, modeled on existing frameworks, to ensure long-term sustainability. Teacher recertification every 2–3 years will preserve instructional quality.

### 6.3 Instructors and institutional capacity

Instructor quality is a critical determinant of training effectiveness. International experiences show that many programs falter because instructors are not adequately trained or certified. India should therefore establish a “train-the-trainer system” in which teachers, physical education instructors, and leaders of the National Cadet Corps or National Service Scheme undergo structured certification.

• To support this model, organizations such as the Indian Resuscitation Council, Indian Heart Rhythm Society, Cardiological Society of India, Indian Academy of Pediatrics, and departments of cardiology, emergency medicine, and anesthesiology within teaching hospitals can serve as primary instructor hubs. Their involvement ensures clinical accuracy, ongoing quality control, and a sustainable pipeline of certified instructors.
• A centralized National Instructor Registry should be created under the Indian Resuscitation Council, ensuring uniform standards and monitoring.
• Instructors must undergo recertification every three years, modeled on existing frameworks, with periodic audits of teaching quality.
• Supported by e-learning modules and hybrid instruction to ensure scalability, particularly in rural areas where access to proposed hubs may be limited.
• Partnerships with American Red Cross, non-governmental organizations, and private sector CSR initiatives can supplement resources while ensuring equity.
• Teachers require certification in CPR instruction via existing AHA or American Red Cross courses. Refresher training should be obtained every three years.
• State departments should monitor and publish annual CPR training compliance reports to track instructor activity and coverage.

### 6.4 AED deployment in educational institutions

AED accessibility must accompany CPR education to maximize survival after sudden cardiac arrest. Deployment should be systematic, socially responsible, and aligned with national public health goals.

• Mandatory installation: Every educational institution should install at least one AED in a clearly visible and accessible location—such as the main entrance, gymnasium, or auditorium—with standardized signage to prevent “AEDs in closets.”
• Hands-on familiarity: Students should routinely practice locating and operating AEDs during CPR sessions to ensure rapid, confident use during emergencies.
• Integration and connectivity: All AEDs should be GPS-tagged and linked to local EMS dispatch systems to enable real-time mapping and coordination during cardiac arrests.
• Registry and accountability: Devices must be incorporated into a national AED registry with regular state-level audits. Each institution should designate a CPR and AED coordinator responsible for quarterly functionality checks and reporting. Noncompliance should prompt corrective oversight and retraining.
• Sustainability and social responsibility: Governments should provide dedicated funding for under-resourced and rural schools, supported by corporate social responsibility (CSR) partnerships and community-based sponsorships. Such initiatives strengthen social ownership of life-saving infrastructure and foster a culture of collective accountability in cardiac arrest preparedness.

### 6.5 Skill retention and innovation

Skill decay is a major challenge and has been demonstrated in several studies previously. To mitigate this, refresher sessions must be built into the program. Data show that students are not only open to but also supportive of recurrent training.^35^ Training should consider the following:

• Low-cost training manikins and AED simulators are essential. Feedback manikins that provide real-time correction on compression depth and rate have been shown to significantly enhance learning outcomes [61].
• Innovative approaches and digital tools, including mobile applications, gamified modules, and virtual or augmented reality platforms, can provide engaging ways to rehearse skills. Virtual and augmented reality modules are particularly valuable in resource-limited settings, as they allow standardized training without high equipment costs. Studies in Europe and Asia demonstrate that gamified, mobile CPR apps improve willingness to perform CPR and increase retention over 6–12 months.
• Short, frequent sessions, lasting only 5–10 min monthly, improve skill retention more effectively than one-time annual workshops.
• India's success with large scale public health engagement, such as the annual celebration of Yoga Day, illustrates how repeated, visible activities can normalize health behaviors. Similar recurring CPR practice days can reinforce skills, increase public participation, and embed lifesaving behavior into the cultural fabric.

### 6.6 Student engagement and amplifying reach

Students are powerful agents of change and act as multipliers of effect. In India, youth enthusiasm should be harnessed by the following means.

• Peer-to-peer reinforcement, such as CPR clubs and training events, create a culture of practice. This has been demonstrated by programs in Singapore and Japan [62].
• Appointing CPR champions within schools and colleges also encourage excellence.
• Encourage students to teach CPR to parents, siblings, and community members as homework.
• Involve the National Cadet Corps, National Service Scheme, and the Bharat Scouts and Guides to expand reach beyond the classroom.
• Annual national student CPR challenge competitions to motivate adoption.
• Establish a national student lifesaver corps, modeled on youth volunteer systems abroad, to formalize student involvement in community training and disaster response
• Public campaigns tied to national CPR days, sports tournaments, or cultural festivals can further normalize lifesaving behaviors.

### 6.7 Funding and accountability strategies

One of the most striking weaknesses identified elsewhere was the absence of funding provisions in many mandates [63]. Without resources for equipment, training aids, and maintenance, programs cannot be sustained. For India, explicit financial commitments must be embedded within legislation. Core funding should come from federal and state governments, with CSP initiatives to supplement the need. Additional considerations should include the following.

• Local manufacturing to reduce costs of manikins and AED trainers, improving scalability.
• CSR contributions should be incentivized through tax credits that support programs, with further incentives for successful implementation and increase in CPR and AED rates, and reductions in OHCA.
• Legislation should earmark dedicated budgets within education and health ministries for CPR/AED programs. This avoids the problem seen in several US states, where schools were required to train students without receiving resources. Florida House Bill 1607 is a notable example where funding and liability protections were written into the law, ensuring sustainability [64].
• Annual reporting should be required from schools and colleges, including numbers of students trained, AED functionality, and refresher drills conducted, as transparent monitoring and public reporting will enhance accountability and promote continuous improvement. A national and publicly available CPR registry should be established to do so. This can be modeled on existing registries in the US and Denmark.
• Random audits of the public and institution -specific registries should be conducted regularly by district education and health inspectors.
• School funding should be tied to compliance and registry outcomes. Failure to comply should result in penalties such as loss of accreditation points or reduced access to central education grants.

### 6.8 Implementation roadmap

In collaboration with professional societies such as the Indian Academy of Pediatrics (IAP), the Indian Heart Rhythm Society (IHRS), Cardiological Society of India (CSI), Indian Society of Electrocardiology (ISE) and other relevant organizations, the State Department of Education should take the lead in coordinating this initiative. To ensure comprehensive implementation, the Department of Health should also be integrated into the framework, leveraging existing resources to create a dedicated unit capable of executing and sustaining the CPR and AED program.

**Fig. 5:**
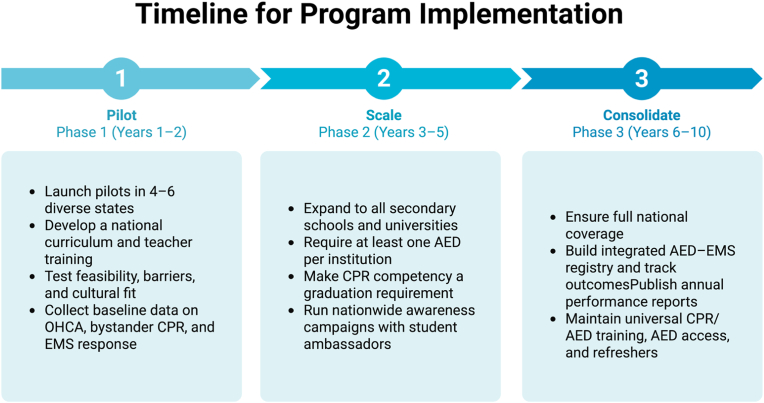
Timeline for national CPR and AED education program implementation. The initiative is structured in three progressive phases. Phase 1 (Pilot, Years 1–2): Launch pilot programs in diverse states, develop standardized curricula, train teachers, and collect baseline data on cardiac arrest and emergency response. Phase 2 (Scale, Years 3–5): Expand to all secondary schools and universities, mandate at least one AED per institution, integrate CPR competency as a graduation requirement, and launch national awareness campaigns. Phase 3 (Consolidate, Years 6–10): Ensure full nationwide coverage, establish AED–EMS registries to track outcomes, and maintain annual CPR/AED training, audits, and public reporting to sustain quality and engagement.

A phased approach is recommended to ensure feasibility and sustainability. We propose a multi-year approach, beginning with pilot programs and evaluation across several regions (Fig. 5). Pilot programs should be launched in selected states, allowing curriculum testing and deployment in controlled settings. Lessons from these pilots can guide national refinement. Each phase should include external evaluation by an independent monitoring board to ensure fidelity of implementation [65]. To accelerate cultural normalization, CPR education should be tied to public campaigns during later phases to reinforce skills and visibility. Towards the end of a third proposed phase, implementation should also address disparities by mandating rural-first funding, mobile simulation units, and multilingual teaching resources.

Existing national and state disaster response teams possess established logistics networks, rapid mobilization capacity, and command structures that can support CPR education rollout. Strengthening these teams through integration with CPR and AED initiatives, joint drills, and shared infrastructure can enhance emergency preparedness and create a unified response framework.

## 7 Implementation challenges

Implementing a nationwide program for CPR and AED training in India will inevitably face financial and logistical challenges. The scale of India's educational system makes program costs a central concern [66]. Additionally, regular retraining requires recurring investment rather than one-time funding. This can only be mitigated with diversified funding streams. Federal and state government will, of course, need to commit baseline support. However, CSR initiatives, partnerships with non-governmental organizations, and philanthropic efforts can provide additional resources; local manufacturing can also reduce reliance on costly imports. Leveraging technology can further lower expenses.

Logistical challenges extend beyond finances and lies in ensuring equity. India's linguistic diversity demands training materials in multiple regional languages. Rural and semi-urban schools may lack infrastructure or trained faculty, necessitating a “train-the-trainer” model supported by regional CPR/AED hubs. Without deliberate planning, urban and private institutions may adopt these programs faster, while rural and government schools lag behind. To prevent widening disparities, funding allocations should prioritize underserved regions and include low-cost mobile training units [31]. Device maintenance represents another major hurdle – AED batteries and pads require replacement every few years, and vandalism or neglect may render devices unusable [67]. Thus, establishing clear lines of accountability linking schools to regional hubs for audits and technical support will be essential for sustainability.

Finally, resistance to curriculum changes and competing educational priorities may slow adoption. Collaboration between the Ministry of Education, teacher unions, and parent associations is essential to ensure acceptance and avoid prior pitfalls. Nevertheless, the financial and logistical challenges of implementing such a program, though significant, are not insurmountable. With coordinated efforts, India can establish a cost-effective and sustainable framework that transforms its educational network into a lifesaving public health infrastructure.

## 8 Conclusions

SCA represents one of India's most urgent and under-recognized public health crises. Despite formidable challenges, the path forward is clear: embedding CPR and AED training within schools and colleges offers an unparalleled opportunity to create a self-renewing generation of lifesavers. This requires addressing multiple factors and is supplemented by the creation of a national registry. With decisive action, India can turn its demographic dividend into a public health shield, demonstrating that school-based training is not just feasible, but transformative, and leading the way for other LMICs.

## Disclosures

None relevant to the topic.

## Declaration of competing interest

The authors declare the following financial interests/personal relationships which may be considered as potential competing interests: Indian Pacing and Electrophysiology Journal - Editorial Board Member If there are other authors, they declare that they have no known competing financial interests or personal relationships that could have appeared to influence the work reported in this paper.
